# Risk of Gastric Cancer among Patients with Newly Diagnosed Ulcerative Colitis: A Nationwide Population-Based Study

**DOI:** 10.3390/jcm12082843

**Published:** 2023-04-13

**Authors:** Hee Man Kim, Jihoon Kim, Hyunil Kim, Soon Chang Park, Jung Kuk Lee, Dae Ryong Kang, Su Young Kim, Hyun-Soo Kim

**Affiliations:** 1 Health Promotion Center, Gangnam Severance Hospital, Yonsei University College of Medicine, Seoul 06273, Republic of Korea; 2 Department of Physiology, Yonsei University Wonju College of Medicine, Wonju 26426, Republic of Korea; 3 Department of Internal Medicine, Yonsei University Wonju College of Medicine, Wonju 26426, Republic of Korea; 4 Department of Biostatistics, Yonsei University Wonju College of Medicine, Wonju 26426, Republic of Korea; 5 Department of Precision Medicine & Biostatistics, Yonsei University Wonju College of Medicine, Wonju 26426, Republic of Korea

**Keywords:** gastric cancer, ulcerative colitis, risk factors

## Abstract

Background: Few studies have investigated the risk of gastric cancer (GC) in ulcerative colitis (UC), and the results have been inconsistent. This study aimed to assess the risk of gastric cancer in newly diagnosed UC patients. Methods: Based on claims data from Korean National Health Insurance from January 2006 to December 2015, we identified 30,546 patients with UC and randomly selected 88,829 non-UC individuals as controls, who were matched by age and sex. Multivariate Cox proportional hazards regression was used to calculate adjusted hazard ratios (HRs) for gastric cancer events, with covariates taken into account. Results: During the study period, a total of 77 (0.25%) patients with UC and 383 (0.43%) non-UC individuals were diagnosed with GC. After multivariable adjustment, the HR for GC was 0.60 (95% CI: 0.47–0.77) in patients with UC, using non-UC individuals as the reference group. When stratified by age, the adjusted HRs for GC in UC patients were 0.19 (95% CI: 0.04–0.98) for those aged 20–39 years at the time of UC diagnosis, 0.65 (95% CI: 0.45–0.94) for 40–59, and 0.60 (95% CI: 0.49–0.80) for ≥60 as compared to non-UC individuals in the corresponding age groups. When stratified by sex, the adjusted HR for GC was 0.54 (95% CI: 0.41–0.73) in male UC patients of all ages. Within UC patients, a multivariable analysis revealed that the HR for GC was 12.34 (95% CI: 2.23–68.16) for those aged ≥ 60 years at the time of diagnosis of UC. Conclusions: Patients with UC had a decreased GC risk compared with non-UC individuals in South Korea. Within the UC population, advancing age (≥60 years) was identified as a significant risk factor for GC.

## 1. Introduction

Inflammatory bowel disease (IBD) is a chronic inflammatory disease of the gastrointestinal tract. Ulcerative colitis (UC) and Crohn’s disease are the main forms of IBD. Usually, UC involves the lower gastrointestinal tract, such as the colon, but not the upper gastrointestinal tract. Interestingly, UC is associated with cancers of the gastrointestinal tract, including colorectal cancer and small bowel cancer [1,2,3]. In particular, patients with UC have an increased risk of colorectal cancer [4,5,6,7,8], and surveillance for colorectal cancer is common in such patients. Chronic inflammation is considered a major risk factor in UC-associated colorectal cancer.

However, the association between gastric cancer (GC) and UC remains unclear [2,9,10,11]. Several population-based studies showed a modest (non-substantial) decrease in the risk of GC in patients with UC [2,10,11]. Furthermore, these studies did not have sufficient GC cases for statistical analysis [2,10,11]. The incidence of UC has been increasing in Korea [12], and the incidence and prevalence of GC is high compared to that in Western countries [13]. Therefore, we assume that the fact that the Korean population has an increasing incidence of UC and a high incidence of GC may be appropriate to investigate the relationship between UC and GC. We conducted this retrospective cohort study using claims data of the Korean National Health Insurance (NHI) database to investigate the risk of GC in patients with newly diagnosed UC.

## 2. Materials and Methods

### 2.1. Study Population

Our retrospective cohort study used claims data from the NHI database, which provided comprehensive information of claims data of beneficiaries of Medical Aid and NHI in South Korea. In 2015, the database contained information on 52,034,424 beneficiaries, including demographics, medical treatment claims for hospitalization and ambulatory care, pharmaceutical prescriptions, medical procedures, and diagnoses based on the International Classification of Diseases, 10th revision (ICD-10) [2].

We based our research methodology on a previously published study [14], using the same research method and analysis but modifying the diagnostic codes. In brief, the study included patients with newly diagnosed UC and matched individuals without UC from the Korean general population.

This retrospective study aimed to investigate the incidence of GC in patients with UC compared to matched individuals without UC from the Korean general population. The study utilized data from beneficiaries of Medical Aid and NHI. Patients with UC diagnosed between January 2004 and December 2015 were initially selected, and a washout period of 2 years (January 2004–December 2005) was applied to exclude prevalent cases [15]. Patients with newly diagnosed UC who had not visited the hospital during the washout period and visited the hospital with a diagnosis of UC between January 2006 and December 2015 were enrolled in the study. Age- and sex-matched individuals in a 3:1 ratio (non-UC: UC) were randomly selected from the NHI database between January 2006 and December 2015, using stratification based on age, sex, residential area, and socioeconomic status. The selection of a 3:1 ratio of non-UC to UC was a deliberate choice to ensure sufficient sample sizes for both groups while maintaining balance between them. Stratification according to age, sex, residential area, and socioeconomic status was divided, and random sampling was performed for each stratification. The age- and sex-matched individuals were defined as non-UC individuals. The primary endpoint was the incidence of GC.

### 2.2. Ethical Considerations

This study was conducted in accordance with the ethical standards of the responsible committee on human experimentation, both institutional and national, and with the Helsinki Declaration of 1964 and later versions. This study was approved by the Institutional Review Board (IRB) of Wonju Severance Christian Hospital, Wonju, Korea (CR312044). Informed consent was exempted by the IRB, as this was a retrospective study.

### 2.3. Definitions

To identify newly diagnosed UC and GC cases for data analysis, we obtained operational definitions from the NHI data, primarily based on insurance claims. Patients with UC were defined as those with the ICD-10 diagnostic code K51 and prescriptions for UC, as validated in a previous study. Prescription for UC was defined as use of steroids for at least 3 months, any use of 5-aminosalicylic acid, any use of immunomodulators such as 6-mercaptopurine, azathioprine, and/or methotrexate, or any use of biologic agents such as tumor necrosis factor (TNF) alpha antagonist [16]. Our operational definition had a sensitivity of 93.1 (91–94.7) and a specificity of 98.1 (96.9–98.8) in the study [16]. Non-UC individuals were those without diagnostic codes for IBD, specifically K51 for UC and K50 for Crohn’s disease.

Patients with GC were defined as those with the ICD-10 diagnostic code of C16, and we use the V code system to increase accuracy and exclude false positive cases of GC. The V code system, developed by the Korean Ministry of Health and Welfare, is used to register patients with cancer and rare and intractable disorders and diseases, and is managed by the NHI to support a significant portion of medical expense for registered patients [17]. In our study, we used the V codes V193–194 to exclude false-positive cases of GC [17]. Individuals included in this cohort were censored at the date of dropout (due to death or emigration) or at the end of follow-up (December 2015) if they did not fulfill the operational definition of GC during the study period.

### 2.4. Statistical Analysis

In this study, we analyzed the association between UC and the risk of GC, adjusting for various covariates, including social economic status, residential area, comorbidities, and the modified Charlson comorbidity index (CCI). The CCI is a weighted index that predicts 1-year mortality in patients with multiple comorbidities and is commonly used in epidemiological studies of IBD [18,19]. In our study, we modified the CCI by excluding cancers from the list of comorbidities. In addition, we included hypertension (ICD-10 codes: I10–13, I15), diabetes mellitus (E78), cerebrovascular disease (I60–69), cardiovascular disease (I20–25, I34–37), and cholangitis (K83) as independent comorbidities. For the analysis, we divided the health insurance quantile into 20 quantiles and defined socioeconomic status by dividing the 20 insurance quantiles into three categories (low, mid, and high).

We included the following as IBD medications: steroids, 5-aminosalicylic acid (5-ASA), immunomodulators, such as 6-mercaptopurine, azathioprine, and/or methotrexate, and biologic agents, such as TNF alpha antagonists. We classified patients as users of a drug if the drug was taken at least once, except for steroids, where only use for at least 3 months was classified as steroid use. We did not consider drug combinations or change in drug types or doses over time. Duration of prescription of 5-ASA was classified into 4 quartiles: the first ranged 0 to 222 days, the second ranged 223 to 931 days, the third ranged 932 to 2016 days, and the fourth was ≥2017 days.

We used multivariate Cox regression models with Firth correction to calculate the hazard ratios (HRs), and evaluate the risk of GC in UC patients, with age- and sex-matched non-UC individuals as reference. The event was defined as the occurrence of GC, and the time-to-event was defined as the time until gastric cancer occurs. We adjusted the HRs for GC by age, sex, socioeconomic status, residential area, and comorbidities, including hypertension, diabetes mellitus, cerebrovascular disease, and cardiovascular disease. Additionally, we used Gray’s test to determine significant differences between UC patients and non-UC individuals.

Continuous variables are presented as the means ± standard deviations and categorical variables are presented as numbers and percentages. We used the Student *t*-test for continuous variables and the chi-square test for binary and categorical variables to compare characteristics between groups. All statistical analyses were two-tailed, and we considered a *p*-value of <0.05 statistically significant. We conducted all analyses using SAS version 9.4 (SAS Institute Inc., Cary, NC, USA).

## 3. Results

### 3.1. Study Population

**Figure 1:**
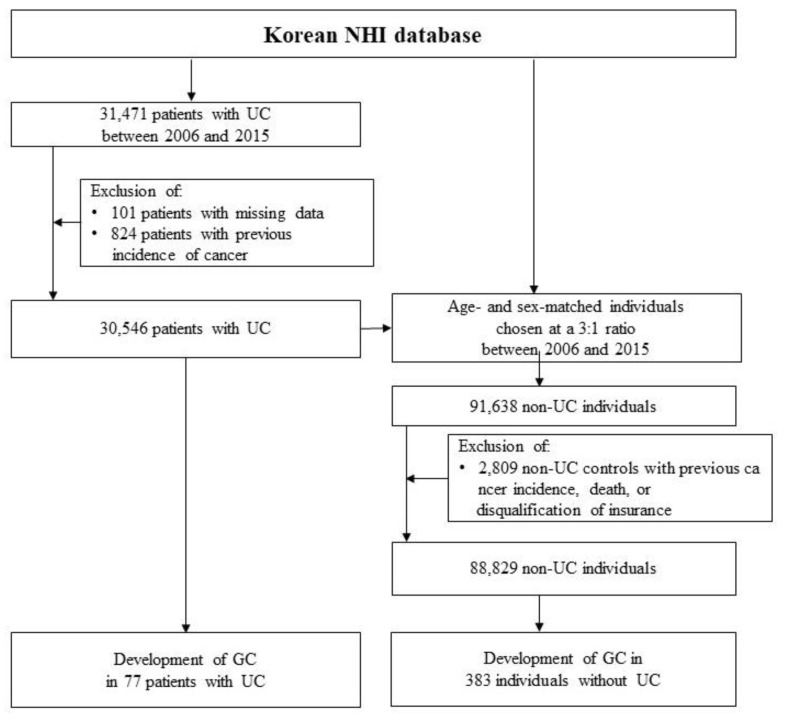
Flow chart of the study population.

The stud population included 31,471 patients who were newly diagnosed with UC between January 2006 and December 2015. Out of these, 925 patients were excluded from the analysis, including 101 with missing data and 824 with a history of cancer. Therefore, a total of 30,546 patients were included in the analysis. For the control group, we selected 91,638 individuals from the general population who were matched by age and sex to the UC patients. Out of these, 2809 individuals were excluded due to disqualification for insurance, a history of cancer, or death. As a result, 88,829 matched individuals were enrolled as non-UC individuals (Figure 1).

The mean follow-up periods for patients with UC and non-UC individuals were 5.11 ± 2.96 and 5.20 ± 2.95 years, respectively. During the study period, among 30,546 patients with UC, 77 were diagnosed with GC, while among 88,829 non-UC individuals, 383 were diagnosed with GC. The crude incidence rate of GC was 48.6 per 100,000 persons per year in patients with UC, and 82.0 per 100,000 persons per year in non-UC individuals.

Of the patients with UC, 58.65% (17,914) were men, which was similar to the proportion of men among non-UC individuals (58.58%). The mean age of patients with UC was 42.7 ± 16.1 years, which was similar to that of non-UC individuals (42.3 ± 16.0 years).

The study found that high socioeconomic status and urban residential area were more prevalent factors among patients with UC than non-UC individuals (*p* < 0.0001). Diabetes mellitus, cerebrovascular disease, cardiovascular disease, and cholangitis were more prevalent among patients with UC than non-UC individuals (Table 1).

### 3.2. Risk of GC in Patients with UC

The HR for GC, adjusted for multiple variables, was 0.60 (95% CI: 0.47–0.77) in patients with UC, with reference to non-UC individuals (Table 2).

To investigate the potential influence of age at UC diagnosis on the unexpected decrease in GC risk among patients with UC, we calculated adjusted HRs stratified by age groups at the time of UC diagnosis. The HRs for GC were 0.19 (95% CI: 0.04–0.98) in those aged 20–39 years at diagnosis of UC compared to non-UC individuals ages 20–39 years, 0.65 (95% CI: 0.45–0.94) in those aged 40–59 years at diagnosis of UC compared to non-UC individuals aged 40–59 years, and 0.60 (95% CI: 0.49–0.80) in those aged ≥ 60 years compared to non-UC individuals aged ≥60 years.

Thereafter, we calculated adjusted HRs stratified by sex. The HRs for GC were 0.54 (95% CI: 0.41–0.73) in male patients with UC compared to male non-UC individuals, and 0.81 (95% CI: 0.51–1.27) in female patients with UC compared to female non-UC individuals. Within male patients with UC, the HRs for GC were 0.51 (95% CI: 0.32–0.81) in those aged 40–59 years at the time of UC diagnosis, and 0.61 (95% CI: 0.42–0.89) in ≥60.

### 3.3. Risk Factors for GC within Patients with UC

To investigate the risk factors for GC among patients with UC, the patients were subdivided into GC and non-GC groups (Table 3). The GC group had a higher proportion of men than the non-GC group, and patients in the GC group received a UC diagnosis at an older age than those in the non-GC group.

After adjusting for covariates, the multivariable analyses showed that patients who were diagnosed with UC at the age of ≥60 years had a considerably higher HR for GC than those who were diagnosed at the age of 0–19 years (HR; 12.34, 95% CI: 2.23–68.16) (Table 4). However, the other variables, including sex, did not show substantial differences between the GC and non-GC groups.

### 3.4. The Impact of 5-ASA on the Risk of GC

We also investigated the impact of 5-ASA on the risk of GC in UC patients (Table 5). Our findings suggested that the 3rd and 4th quartile duration of 5-ASA usage duration were strongly associated with reduced HRs, 0.44 (95% CI: 0.22–0.87), and 0.42 (95% CI: 0.24–0.75), respectively, compared to the 1st quartile. These results indicate that long-term use of 5-ASA can lower the risk of gastric cancer in UC patients.

## 4. Discussion

Our nationwide cohort study, included 30,546 patients with UC, found that 77 (0.25%) of these patients developed GC over a 10-year period. We also found that patients with UC had a significantly lower risk of developing GC compared to non-UC individuals (HR; 0.60, 95% CI: 0.47–0.77) with multivariable adjustment. To the best of our knowledge, our study is the first research to demonstrate the strong association between UC and the risk of GC. Other studies showed the low risk of gastric cancer in patients with UC, but the risk was statistically not significant. One meta-analysis showed that the risk of GC was low in patients with UC, but the finding was not substantial (odds ratio [OR]: 0.79, 95% CI: 0.51–1.07), and when stratified by geographical area, Asia had a lower OR compared to North America and Europe, but not a significant one [20]. A Korean study reported a standardized incidence ratio (SIR) of 0.70 (95% CI: 0.3–1.37) for GC in UC patients compared to the general population in South Korea, but, again, not significant [2]. An Asian study conducted in Hong Kong which included 1603 UC patients reported a SIR of 0.24 (95% CI: 0.03–1.74) for GC compared to the general population [11].

The reason for selecting a 1:3 of UC: non-UC ratio instead of 1:1 was intended to achieve a sufficient sample size of both groups for statistical analysis while still maintaining a reasonable balance between the two groups. A larger sample size increases the statistical power. Additionally, a larger control group provides a more representative sample of the general population. The 1:3 ratio is a commonly used ratio in epidemiological studies [14].

According to data from the Korea Central Cancer Registry, the age-standardized incidence rate of gastric cancer in South Korea decreased from 46.7 per 100,000 population in 2006 to 32.7 per 100,000 population in 2015 [21]. According to data from the National Health Insurance Service and the Korean Association for the Study of Intestinal Diseases, the age- and sex-standardized incidence rate of ulcerative colitis in South Korea increased from 5.4 per 100,000 population in 2010 to 7.3 per 100,000 population in 2015 [22].

The disease of UC does not directly affect the organ of the stomach, and so the reason for the decreased risk of GC in UC remains unclear. Other factors, such as *H. pylori* infection, are suggested to play a role in the relationship between UC and GC [23,24,25]. *H. pylori* is a risk factor for GC, and the prevalence of *H. pylori* is associated with up to 80% of sporadic GC [23,24]. The prevalence of *H. pylori* is low in patients with UC [26,27,28]. This low prevalence of *H. pylori* may result from the following: (1) medications for treatment of UC may eradicate *H. pylori*, and (2) *H. pylori* may be protective against the development of UC by altering the host’s immunologic response toward increased T-regulatory cell immune response and inflammation suppression. However, in our study, the analysis of *H. pylori* status was not performed because claims data from the Korean NHI did not have the data for *H. pylori* status.

Our study showed that, when stratified by sex, male patients with UC had a decreased risk of GC compared to non-UC male individuals. However, among patients with UC, female patients with UC had a decreased risk for GC, as male sex is generally a risk factor for sporadic GC [13].

In addition, when stratified by age at diagnosis of UC, those with 20–39, 40–59, ≥60 years had a lower relative risk compared to non-UC individuals of the same age. A young age (0–19 years) at diagnosis of UC was not substantially associated with GC risk compared to non-UC individuals of the same age. This may be caused by the low incidence rate of GC rather than a true lack of relationship [13]. However, among patients with UC, old age (≥60 years) was associated with an increased risk of GC compared to young age (0–19 years), which was consistent with sporadic GC. Similarly, one study showed that age was a significant risk factor for GC in UC (OR: 1.068, 95% CI: 1.022–1.117) [29].

In our study, we investigated the effect of 5-ASA on GC in patients with UC, showing that long-term use of 5-aminosalicylic acid was strongly associated with a decreased risk of GC in patients with UC. However, there have been few studies on the effect of 5-ASA on gastric cancer, and, to our knowledge, our study is the first to demonstrate that long-term use of 5-ASA reduces the risk of gastric cancer in patients with UC. We speculate that the effects of 5-asa on gastric cancer may have been shown through several pathways involving inhibition of colorectal cancer by 5-ASA. 5-ASA is a drug class used for remission induction and maintenance of remission in UC, and epidemiological studies suggest that 5-ASA is associated with a lower risk of developing colitis-associated colorectal cancer [30]. The higher dose of 5-ASA may be a better preventive for colorectal cancer. 5-ASA has a chemo-preventive effect via various pathways [31]. The mechanism involving association between 5-ASA and GC remains to be clarified.

Other medications including steroids, immunomodulators, and biologics are used for management of UC. However, other medications were not used for multivariate adjustment. Regarding steroids, it was difficult to determine the duration of use, since they are used temporarily, and they are used often in severe cases, which may lead to bias. Biologics are relatively new drugs in use, which may also result in bias. Additionally, these medications are often used in combination, which makes analysis difficult. As a result, we only analyzed the most commonly used medication, 5-ASA.

Our results could be applied to studies related to the mechanism of occurrence of gastric cancer. In Korea, the incidence of gastric cancer is very high. UC is associated with an increased risk of various cancers, such as colorectal cancer. However, our study concluded that, contrary to our expectations, the risk of gastric cancer in UC patients was lower than that of controls in Korea. UC is caused by an abnormality in the immune system, which may be a pathway that lowers the occurrence of gastric cancer.

One clinical application may be as follows. Within a UC population, patients with old age (≥60 years) at diagnosis have an increased risk of gastric cancer. These findings suggest that UC patients with old age at diagnosis should be considered to receive upper gastrointestinal endoscopy for screening regularly.

Our study has some limitations, mainly related to the nature of the claims data of the NHI database. First, we used an operational definition for the diagnosis of UC. However, since most IBD patients in Korea are diagnosed and treated at tertiary hospitals by IBD specialists, the ICD codes of UC were considered accurate [12]. Furthermore, we included the prescription history of IBD-specific medications to enhance the diagnostic accuracy of UC. Second, clinical data on UC, such as disease severity and extent that could have influenced the risk of GC, were not available. Third, we did not have information on potential confounding factors linked to the risk of GC in UC patients, such as H. pylori infection, smoking, alcohol consumption, or familial history of cancer. Forth, the number of GC cases was limited, which may have weakened the robustness of our findings, and the conclusions should be interpreted with caution. Finally, our study did not exclude precancerous lesions, including atrophic gastritis, intestinal metaplasia, and dysplasia, which may have influenced our results. These limitations should be taken into account in future prospective studies.

## 5. Conclusions

In our study, patients with incident UC had a lower risk of developing GC compared to non-UC individuals in Korea. Our study also found that patients with old age at diagnosis UC and of the male gender were associated with a decreased risk of GC compared to non-UC individuals in the same age groups. In subgroup analysis within patients with UC, old age at diagnosis (40–59 years and ≥60 years) and male gender were increased risk factors for GC. Based on these findings, it is recommended that UC patients who are old (40–59 years and ≥60 years) at diagnosis of UC should receive upper gastrointestinal endoscopy for screening. It is important to note that due to the small incidence of gastric cancer and the limitations of the claim data itself, the results should be interpreted with caution, and further studies are needed to confirm these findings.

## Figures and Tables

**Table 1:** Baseline characteristics of the study population including UC patients and controls.

Characteristic	UC Patients	Non-UC Individuals	*p* Value
Total	30,546	88,829	
Person-year	158,380	467,232	
Gastric cancer	77 (0.25%)	383 (0.43%)	<0.0001
Person-year	291	1413	
Incidence rate (1/100,000 person-years)	48.6	82.0	
Sex (male)	17,914 (58.65)	52,032 (58.58)	0.8291
Age at diagnosis of UC *			
Mean ± SD	42.7 ± 16.1	42.3 ± 16.0	<0.0001
0–19	2021 (6.62)	6055 (6.82)	0.0010
20–39	11,646 (38.13)	34,450 (38.78)	
40–59	11,848 (38.79)	34,515 (38.86)	
≥60	5031 (16.47)	13,809 (15.55)	
Social economic status			<0.0001
Low	7218 (23.63)	22,141 (24.93)	
Mid	8934 (29.25)	32,129 (36.17)	
High	14,394 (47.12)	34,559 (38.91)	
Residential area			<0.0001
Urban	28,099 (91.99)	78,266 (88.11)	
Rural	2424 (7.94)	9274 (10.44)	
Comorbidities			
HTN	8548 (27.98)	24,417 (27.49)	0.0942
DM	7694 (25.19)	19,408 (21.85)	<0.0001
Cerebral vascular disease	502 (1.64)	969 (1.09)	<0.0001
Cardiovascular disease	504 (1.65)	935 (1.05)	<0.0001
Cholangitis	652 (2.13)	846 (0.95)	<0.0001
CCI without tumor factor			<0.0001
0	8891 (29.11)	31,466 (35.42)	
1	10,203 (33.40)	28,827 (32.45)	
2	6016 (19.69)	15,188 (17.10)	
≥3	5436 (17.80)	13,348 (15.03)	
IBD medication			
Steroid	14,170 (46.39)	5801 (6.53)	<0.0001
5-ASA	30,285 (99.15)	25 (0.03)	<0.0001
Thiopurines	5181 (16.96)	84 (0.09)	<0.0001
Biologics	1579 (5.17)	30 (0.03)	<0.0001
Follow-up period (years)	5.11 ± 2.96	5.20 ± 2.95	<0.0001

Data are presented as mean ± SD or number (%). UC = ulcerative colitis, IBD = inflammatory bowel disease, CCI = Charlson Comorbidity Index, HTN = hypertension, DM = diabetes mellitus, CVD = cardiovascular disease, 5-ASA = 5-aminosalicylic acid. * In non-UC group, “age” was defined as the matched age to the UC patients when selected.

**Table 2:** Adjusted Hazard ratios of gastric cancer stratified by sex and age in patients with incident UC between January 2006 and December 2015.

Age	Age at Diagnosis (Year)	GC Cases	Adjusted HR ^a^	95% CI	*p* Value
Age groups	All	77	0.60	0.47–0.77	<0.0001
	0–19 years	1	5.81	0.05–714.65	0.473
	20–39 years	1	0.19	0.04–0.98	0.047
	40–59 years	35	0.65	0.45–0.94	0.021
	≥60 years	40	0.60	0.49–0.80	0.004
**Sex**	**Age at Diagnosis (year)**	**GC Cases**	**Adjusted HR ^a^**	**95% CI**	***p* Value**
Male	All	54	0.54	0.41–0.73	<0.001
	0–19 years	1	4.99	0.05–1.54	0.4893
	20–39 years	0	-	-	-
	40–59 years	21	0.51	0.32–0.81	0.0045
	≥60 years	32	0.61	0.42–0.89	0.0111
Female	All	23	0.81	0.51–1.27	0.3559
	0–19 years	0	-	-	-
	20–39 years	1	0.49	0.08–2.89	0.4277
	40–59 years	14	1.12	0.61–2.06	0.7232
	≥60 years	8	0.62	0.29–1.31	0.2089

UC = ulcerative colitis, HR = hazard ratio, GC = gastric cancer. ^a^ Multivariable adjustment for age, sex, social economic status, residential area, hypertension, diabetes mellitus, cerebrovascular disease, and cardiovascular disease.

**Table 3:** Characteristics of UC patients with/without gastric cancer between January 2006 and December 2015.

Characteristic	UC Patients		
	Gastric Cancer	Non-Gastric Cancer	*p* Value
Total number	77	30,469	
Person-years	291	258,089	
Sex (male)	54 (70.13)	17,860 (58.62)	0.0405
Age at diagnosis of UC			
Mean ± SD	59.73 ± 12.04	42.66 ± 16.11	<0.0001
0–19 years	1 (1.30)	2020 (6.63)	<0.0001
20–39 years	1 (1.30)	11,645 (38.22)	
40–59 years	35 (45.45)	11,813 (38.77)	
≥60 years	40 (51.95)	4991 (16.38)	
Social economic status			0.7381
Low	19 (24.68)	7199 (23.63)	
Mid	25 (32.47)	8909 (29.24)	
High	33 (42.86)	14,361 (47.13)	
Residential area			0.7237
Urban	35 (45.45)	14,421 (47.37)	
Suburban	34 (44.16)	13,609 (44.70)	
Rural	8 (10.39)	2416 (7.94)	
Comorbidities			
Hypertension	40 (51.95)	8508 (27.92)	<0.0001
Diabetes Mellitus	32 (41.56)	7662 (25.15)	0.0009
Cerebral vascular disease	2 (2.60)	500 (1.64)	0.3618
Cardiovascular disease	4 (5.19)	500 (1.64)	0.0386
Cholangitis	3 (3.90)	649 (2.13)	0.2268
Modified CCI score ^1^			<0.0001
0	9 (11.69)	8882 (29.15)	
1	14 (18.18)	10,189 (33.44)	
2	28 (36.36)	5988 (19.65)	
≥3	26 (33.77)	5410 (17.76)	
IBD medication			
Steroid	39 (50.65)	14,131 (46.38)	0.4931
5-ASA	75 (97.40)	30,210 (99.15)	0.1407
Thiopurines	6 (7.79)	5175 (16.98)	0.0321
Biologics	1 (1.30)	1578 (5.18)	0.1898
Follow-up period (years)	3.78 ± 2.83	5.19 ± 2.95	<0.0001

Data are presented as mean ± SD or number (%). UC = ulcerative colitis, IBD = inflammatory bowel disease, CCI = Charlson Comorbidity Index, 5-ASA = 5-aminosalicylic acid. ^1^ Modified CCI score was defined as CCI score without tumor factor.

**Table 4:** Multivariate analysis of risk factors for gastric cancer in UC patients.

	UC		
	Adjusted HR ^1^	95% CI	*p* Value
Sex			
Male	1		
Female	0.51	0.32–0.84	0.0077
Age at diagnosis			
0–19 years	1		
20–39 years	0.15	0.02–1.56	0.1125
40–59 years	3.90	0.72–21.04	0.1133
≥60 years	12.34	2.23–68.16	0.0040
Social economic status			
Low	1		
Mid	1.18	0.65–2.14	0.5959
High	0.78	0.44–1.37	0.3779
Residential area			
Urban	1		
Suburban	0.99	0.62–1.59	0.9688
Rural	0.93	0.43–1.98	0.8450
Comorbidities			
Hypertension	0.90	0.54–1.49	0.6667
Diabetes Mellitus	0.92	0.57–1.29	0.7292
Cerebral vascular disease	0.75	0.21–2.71	0.660
Cardiovascular disease	1.58	0.59–4.19	0.3625
Cholangitis	1.79	0.61–5.25	0.2920

UC = ulcerative colitis, HR = hazard ratio. ^1^ Multivariable adjustment for age, sex, social economic status, residential area, hypertension, diabetes mellitus, cerebrovascular disease, cardiovascular disease, and cholangitis.

**Table 5:** Risk of gastric cancer by duration of 5-ASA use in UC patients.

Duration of Medication Use	UC			
	GC Cases	Adjusted HR ^a^	95% CI	*p* Value
5-ASA				
1st quartile	30	1		
2nd quartile	15	0.74	0.39–1.38	0.3373
3rd quartile	12	0.44	0.22–0.87	0.0186
4th quartile	20	0.42	0.24–0.75	0.0033

UC = ulcerative colitis, HR = hazard ratio, GC = gastric cancer, 5-ASA = 5-aminosalicylic acid. ^a^ Multivariable adjustment for age, sex, social economic status, residential area, hypertension, diabetes mellitus, cerebrovascular disease, cardiovascular disease, and cholangitis.

## Data Availability

Not applicable.

## References

[1] Vavricka S.R., Brun L., Ballabeni P., Pittet V., Prinz Vavricka B.M., Zeitz J., Rogler G., Schoepfer A.M. (2011). Frequency and risk factors for extraintestinal manifestations in the Swiss inflammatory bowel disease cohort. Am. J. Gastroenterol..

[2] Jung Y.S., Han M., Park S., Kim W.H., Cheon J.H. (2017). Cancer risk in the early stages of inflammatory bowel disease in Korean patients: A nationwide population-based study. J. Crohn’s Colitis.

[3] Pedersen N., Duricova D., Elkjaer M., Gamborg M., Munkholm P., Jess T. (2010). Risk of extra-intestinal cancer in inflammatory bowel disease: Meta-analysis of population-based cohort studies. Am. J. Gastroenterol..

[4] Andersen N.N., Jess T. (2013). Has the risk of colorectal cancer in inflammatory bowel disease decreased?. World J. Gastroenterol..

[5] Lutgens M.W., van Oijen M.G., van der Heijden G.J., Vleggaar F.P., Siersema P.D., Oldenburg B. (2013). Declining risk of colorectal cancer in inflammatory bowel disease: An updated meta-analysis of population-based cohort studies. Inflamm. Bowel Dis..

[6] Eaden J.A., Abrams K.R., Mayberry J.F. (2001). The risk of colorectal cancer in ulcerative colitis: A meta-analysis. Gut.

[7] Jess T., Rungoe C., Peyrin-Biroulet L. (2012). Risk of colorectal cancer in patients with ulcerative colitis: A meta-analysis of population-based cohort studies. Clin. Gastroenterol. Hepatol..

[8] Jess T., Simonsen J., Jorgensen K.T., Pedersen B.V., Nielsen N.M., Frisch M. (2012). Decreasing risk of colorectal cancer in patients with inflammatory bowel disease over 30 years. Gastroenterology.

[9] Bernstein C.N., Blanchard J.F., Kliewer E., Wajda A. (2001). Cancer risk in patients with inflammatory bowel disease: A population-based study. Cancer.

[10] Jussila A., Virta L.J., Pukkala E., Farkkila M.A. (2013). Malignancies in patients with inflammatory bowel disease: A nationwide register study in Finland. Scand. J. Gastroenterol..

[11] So J., Tang W., Leung W.K., Li M., Lo F.H., Wong M.T.L., Sze A.S.F., Leung C.M., Tsang S.W.C., Shan E.H.S. (2017). Cancer risk in 2621 Chinese patients with inflammatory bowel disease: A population-based cohort study. Inflamm. Bowel Dis..

[12] Yang S.K., Yun S., Kim J.H., Park J.Y., Kim H.Y., Kim Y.H., Chang D.K., Kim J.S., Song I.S., Park J.B. (2008). Epidemiology of inflammatory bowel disease in the Songpa-Kangdong district, Seoul, Korea, 1986–2005: A KASID study. Inflamm. Bowel Dis..

[13] Eom B.W., Jung K.W., Won Y.J., Yang H., Kim Y.W. (2018). Trends in gastric cancer incidence according to the clinicopathological characteristics in Korea, 1999–2014. Cancer Res. Treat..

[14] Kim H.M., Kim J.H., Lee J.K., Kang D.R., Kim H., Kim S.Y., Kim H.S. (2021). Age- and sex-specific risk of colorectal cancer in incident ulcerative colitis during the first 10 years after diagnosis: A nationwide population-based study. Scand. J. Gastroenterol..

[15] Kwak M.S., Cha J.M., Lee H.H., Choi Y.S., Seo S.I., Ko K.J., Park D.I., Kim S.H., Kim T.J. (2019). Emerging trends of inflammatory bowel disease in South Korea: A nationwide population-based study. J. Gastroenterol. Hepatol..

[16] Lee C.K., Ha H.J., Oh S.J., Kim J.W., Lee J.K., Kim H.S., Yoon S.M., Kang S.B., Kim E.S., Kim T.O. (2020). Nationwide validation study of diagnostic algorithms for inflammatory bowel disease in Korean National Health Insurance Service database. J. Gastroenterol. Hepatol..

[17] Hwang Y.J., Kim N., Yun C.Y., Yoon H., Shin C.M., Park Y.S., Son I.T., Oh H.K., Kim D.W., Kang S.B. (2018). Validation of administrative big database for colorectal cancer searched by international classification of disease 10th codes in Korean: A retrospective big-cohort study. J. Cancer Prev..

[18] Charlson M.E., Pompei P., Ales K.L., MacKenzie C.R. (1987). A new method of classifying prognostic comorbidity in longitudinal studies: Development and validation. J. Chronic Dis..

[19] Quan H., Sundararajan V., Halfon P., Fong A., Burnand B., Luthi J.C., Saunders L.D., Beck C.A., Feasby T.E., Ghali W.A. (2005). Coding algorithms for defining comorbidities in ICD-9-CM and ICD-10 administrative data. Med. Care.

[20] Wan Q., Zhao R., Xia L., Wu Y., Zhou Y., Wang Y., Cui Y., Shen X., Wu X.T. (2021). Inflammatory bowel disease and risk of gastric, small bowel and colorectal cancer: A meta-analysis of 26 observational studies. J. Cancer Res. Clin. Oncol..

[21] Jung K.-W., Won Y.-J., Kong H.-J., Lee E.S. (2018). Cancer statistics in Korea: Incidence, mortality, survival, and prevalence in 2015. Cancer Res. Treat. Off. J. Korean Cancer Assoc..

[22] 2020 Inflammatory Bowel Disease Fact Sheet in Korea. http://m.kasid.org/file/IBD%20fact%20sheet_1217.pdf.

[23] Correa P. (1992). Human gastric carcinogenesis: A multistep and multifactorial process--First American Cancer Society Award Lecture on Cancer Epidemiology and Prevention. Cancer Res..

[24] Huang J.Q., Sridhar S., Chen Y., Hunt R.H. (1998). Meta-analysis of the relationship between Helicobacter pylori seropositivity and gastric cancer. Gastroenterology.

[25] Takada K. (2000). Epstein-Barr virus and gastric carcinoma. Mol. Pathol..

[26] Papamichael K., Konstantopoulos P., Mantzaris G.J. (2014). Helicobacter pylori infection and inflammatory bowel disease: Is there a link?. World J. Gastroenterol..

[27] Song M.J., Park D.I., Hwang S.J., Kim E.R., Kim Y.H., Jang B.I., Lee S.H., Ji J.S., Shin S.J. (2009). The prevalence of Helicobacter pylori infection in Korean patients with inflammatory bowel disease, a multicenter study. Korean J. Gastroenterol..

[28] Wu X.W., Ji H.Z., Yang M.F., Wu L., Wang F.Y. (2015). Helicobacter pylori infection and inflammatory bowel disease in Asians: A meta-analysis. World J. Gastroenterol..

[29] Nissen L.H., Assendorp E.L., van der Post R.S., Derikx L.A., de Jong D.J., Kievit W., Pierik M., van den Heuvel T., Verhoeven R., Overbeek L.I. (2016). Impaired gastric cancer survival in patients with inflammatory bowel disease. J. Gastrointest. Liver Dis..

[30] Shah S.C., Itzkowitz S.H. (2022). Colorectal cancer in inflammatory bowel disease: Mechanisms and management. Gastroenterology.

[31] Hsiao S.W., Yen H.H., Chen Y.Y. (2022). Chemoprevention of colitis-associated dysplasia or cancer in inflammatory bowel disease. Gut Liver.

